# Expansion of attentional scope modulates postural control, motor strategies, and attentional network connectivity in healthy adults: a proof-of-concept mixed-methods study

**DOI:** 10.3389/fresc.2026.1758682

**Published:** 2026-03-12

**Authors:** Keisuke Goto, Rui Watanabe, Satoshi Yamamoto, Vivian Sihan Lim, Ryusuke Shimada, Hironobu Kuruma, Atsushi Senoo, Yumi Ikeda

**Affiliations:** 1 Department of Physical Therapy, School of Health Sciences at Narita, International University of Health and Welfare, Chiba, Japan; 2 Department of Physical Therapy, Graduate School of Human Health Sciences, Tokyo Metropolitan University, Tokyo, Japan; 3 Turku PET Centre and Turku University Hospital, University of Turku, Turku, Finland; 4 Department of Physical Therapy, School of Health Sciences, Ibaraki Prefectural University of Health Sciences, Ibaraki, Japan; 5 Department of Rehabilitation, Tokyo Women’s Medical University, Adachi Medical Center, Tokyo, Japan; 6 Department of Radiological Sciences, Graduate School of Human Health Sciences, Tokyo Metropolitan University, Tokyo, Japan

**Keywords:** attentional scope, mixed-methods study, postural control, resting-state fMRI, salience network, ventral attention network

## Abstract

**Introduction:**

Plantar sensory input plays a key role in postural control. However, training protocols that solely amplify this bottom-up input have demonstrated inconsistent efficacy. We hypothesized that a top-down protocol using plantar sensations as a perceptual anchor and expanding the attentional scope from localized plantar sensations to a whole-body reference frame would yield greater improvements than sensory discrimination alone.

**Methods:**

Forty-eight healthy adults (*N* = 48) participated in a single 10-minute session of either Sensory Discrimination Only (SDO) or Sensory Discrimination with Expansion of Attentional Scope (SDE). The SDE protocol employs a brief therapeutic dialogue to facilitate this expansion. The Index of Postural Stability (IPS) was assessed at baseline (T0), immediately after the training (T1), and 30 min after (T2). Semi-structured interviews at T0/T1 were text-mined to quantify motor strategies. Resting-state functional magnetic resonance imaging (rs-fMRI) data were collected at T0/T1 for region-of-interest (ROI)-to-ROI connectivity analyses, focusing on major large-scale brain networks.

**Results:**

The SDE group demonstrated a significant IPS improvement (*Δ*IPS ≈ + 0.09, dz = 0.42) and maintained this improvement at 30 min (T0 vs. T2: dz = 0.32), whereas the SDO group demonstrated no change. Qualitative analyses of self-reported motor strategies in the SDE group indicated attentional expansion beyond a plantar perceptual anchor toward whole-body alignment, reflected by increased references to the shoulders while foot-related references remained common. In rs-fMRI, a cluster within attentional circuitry, including the salience and ventral attention networks, demonstrated a significant group × time interaction [threshold-free cluster enhancement [TFCE]/family-wise error [FWE]-corrected *p* < .05], characterized by reduced connectivity following SDE and a trend toward increased connectivity following SDO.

**Conclusions:**

In this proof-of-concept study, expanding attentional scope from a plantar perceptual anchor toward a whole-body reference frame was associated with immediate, group-level changes across measures. Postural stability improved, alongside changes in self-reported motor strategies and resting-state connectivity within attentional circuitry. Enhancing sensitivity to bottom-up plantar input remains fundamental; however, these findings suggest a potential next step—learning how to interpret and use plantar input as a whole-body reference signal for balance regulation. Confirmation in randomized and longitudinal studies, including evaluation in clinical populations, is warranted.

## 1 Introduction

Human postural control is a complex multisensory integration process. The central nervous system integrates visual, vestibular, and somatosensory information to continually adjust postural responses to task and environmental demands. Sensory input from the plantar surface, serving as the interface between the body and support surface, plays a critical role in body sway detection and appropriate postural response generation. Reducing plantar sensation through targeted anesthesia or cooling significantly impairs balance control (1, 2), and clinically, reduced plantar sensation in older adults is associated with impaired balance and gait instability (3, 4). Collectively, these findings indicate that plantar sensory input is fundamental to postural control and represents a key target for intervention design.

Interventions targeting the plantar surface to improve postural control can be classified by their underlying mechanisms: (1) Peripheral sensory enhancement approaches, including weak electrical stimulation, vibratory stimulation, and textured insoles, reportedly modulate receptor detection thresholds or firing patterns to improve standing balance and postural responses (5–7); (2) Active perceptual learning approaches, which involve repeating discrimination tasks (e.g., distinguishing stiffness or weight) to enhance perceptual acuity, thereby enhancing two-point discrimination and reducing sway (8, 9); (3) Augmented feedback methods, which translate information such as the center of pressure (COP) into visual or auditory cues, enabling participants to update their internal models while actively regulating their sway (10, 11). These approaches have demonstrated efficacy in postural control improvement, thereby supporting the use of plantar-focused sensory interventions as a viable therapeutic option.

However, many conventional interventions for postural control have primarily targeted plantar sensory input, emphasizing stimulus-driven, bottom-up attention. In contrast, postural control is a whole-body process that requires the coordinated regulation of body segments to maintain overall stability (12). This whole-body coordination relies on both automatic brainstem-level processes and goal-directed, top-down attentional control (e.g., constructing motor programs based on an internal representation of the body, i.e., the body schema) (13, 14). Previous approaches have not addressed how bottom-up sensory input and top-down control interact to support whole-body coordination. Motor learning studies report that directing attention locally (e.g., on the feet) increases the attentional demands and leads to greater balance errors (15). Therefore, it may be necessary to broaden the attentional scope while still using plantar sensory input as a perceptual anchor for postural control. Rather than privileging either bottom-up or top-down processing, an optimal strategy may involve training individuals to start from a clear awareness of foot sensations and then extend their attention to a whole-body reference frame. These considerations motivate the present evaluation of an integrative training program that builds from this plantar-sensory anchoring while encouraging the expansion of attentional scope.

Furthermore, assessing behavioral outcomes alone may be insufficient to elucidate the mechanisms underlying such integrative training. Improvements in postural control occur at multiple levels, encompassing changes in motor strategies, conscious regulation of body movements, and the associated neural substrates. However, these subjective dimensions are difficult to access quantitatively. While standardized quantitative metrics are essential, they are limited in their ability to capture the complexity of participants’ subjective experiences (16). To address this gap, qualitative approaches that directly access subjective experience through interviews are valuable for clarifying intervention mechanisms (17–20). Our previous study demonstrated that such subjective experiences could be systematically assessed by using a text-mining approach to qualitatively analyze self-reported motor strategies (21). Therefore, integrating a qualitative approach with objective postural control assessment is crucial for a comprehensive understanding of the intervention's effects. Despite these advances, the neural substrates that mediate these adaptive processes remain poorly understood. Resting-state functional magnetic resonance imaging (rs-fMRI) can capture short-term reconfiguration of large-scale brain networks after recent sensorimotor experience (22–24). Here, we used rs-fMRI to assess lingering network-state changes following training, rather than to infer durable neuroplasticity from a single brief session. We integrated behavioral outcomes, self-reported motor strategies, and post-training resting-state activity within a convergent mixed-methods design. To our knowledge, this unified approach has not been applied to plantar sensory interventions.

Therefore, as a proof-of-concept study in healthy adults, we compared plantar sensory discrimination training only (SDO) with sensory discrimination training combined with expansion of attentional scope (SDE) using a convergent three-level framework: postural stability, self-reported motor strategies, and rs-fMRI. We hypothesized, compared with SDO, SDE would be associated with greater immediate improvements in postural stability, strategy reports indicating attentional expansion beyond a plantar perceptual anchor toward whole-body strategy elements, and post-training differences in functional connectivity (FC) within and between large-scale brain networks. In this context, we interpreted rs-fMRI as a window into short-lived network-state modulation following recent sensorimotor experience, which may reflect carryover of the attentional strategy used during training. We focused on FC within and among four networks central to somatosensory processing, attention, and cognitive control, including the salience network (SN), the frontoparietal network (FPN), the default mode network (DMN), and the sensorimotor network (SMN). We also aimed to provide a multilevel understanding of a training that leverages top-down attentional control to shape the link between somatosensory integration and motor action through the integration and analysis of quantitative and qualitative data. This approach extends beyond a purely sensorimotor perspective by elucidating how changes in conscious motor strategies, behavior, and resting-state brain networks may jointly accompany postural control enhancement.

## 2 Materials and methods

### 2.1 Study design

This study employed a quasi-experimental, comparative design to assess two plantar sensory training protocols: SDO and SDE protocols. A convergent parallel mixed-methods design was adopted, in which quantitative data (postural stability indices and rs-fMRI) and qualitative data (self-reported motor strategies) were collected within the same session, analyzed independently, and integrated for the final interpretation phase. Participants were sequentially allocated in order of enrollment using alternating allocation within sex strata (men/women) to ensure sex balance across groups. Random sequence generation and allocation concealment were not applied.

To maintain procedural consistency, a single physical therapist (K.G.) was responsible for all the training protocols, measurements, and interviews. Assessor and interviewer blinding was not applied due to the nature of the training. This study adhered to the ethical principles of the Declaration of Helsinki and was approved by the Ethics Committee of Tokyo Metropolitan University (Approval No. 18112). Prior to participation, written informed consent was obtained from all participants.

### 2.2 Participants

A total of 50 healthy adults were recruited through on-campus advertisements to participate in this study. The inclusion criteria were as follows: (1) healthy adults aged 20–39 years and (2) right-handedness, as determined by the Edinburgh Handedness Inventory (25) with a Laterality Quotient (LQ) greater than +40. The exclusion criteria were as follows: (1) history of neurological or orthopedic disorders and (2) contraindications to MRI (e.g., implanted metal or claustrophobia).

An *a priori* sample size analysis was performed using G*Power (v3.1) (26) to determine the interaction effect in a 2 (group) × 3 (time) mixed-design analysis of variance (ANOVA), which was the primary statistical test for this study. Parameters were set as follows: effect size (f) = 0.25, *α* = 0.05, statistical powe*r* = 0.95, and correlation among repeated measures=0.5. This calculation indicated a required sample size of 44, and 50 participants were recruited to account for potential attrition.

**Figure 1:**
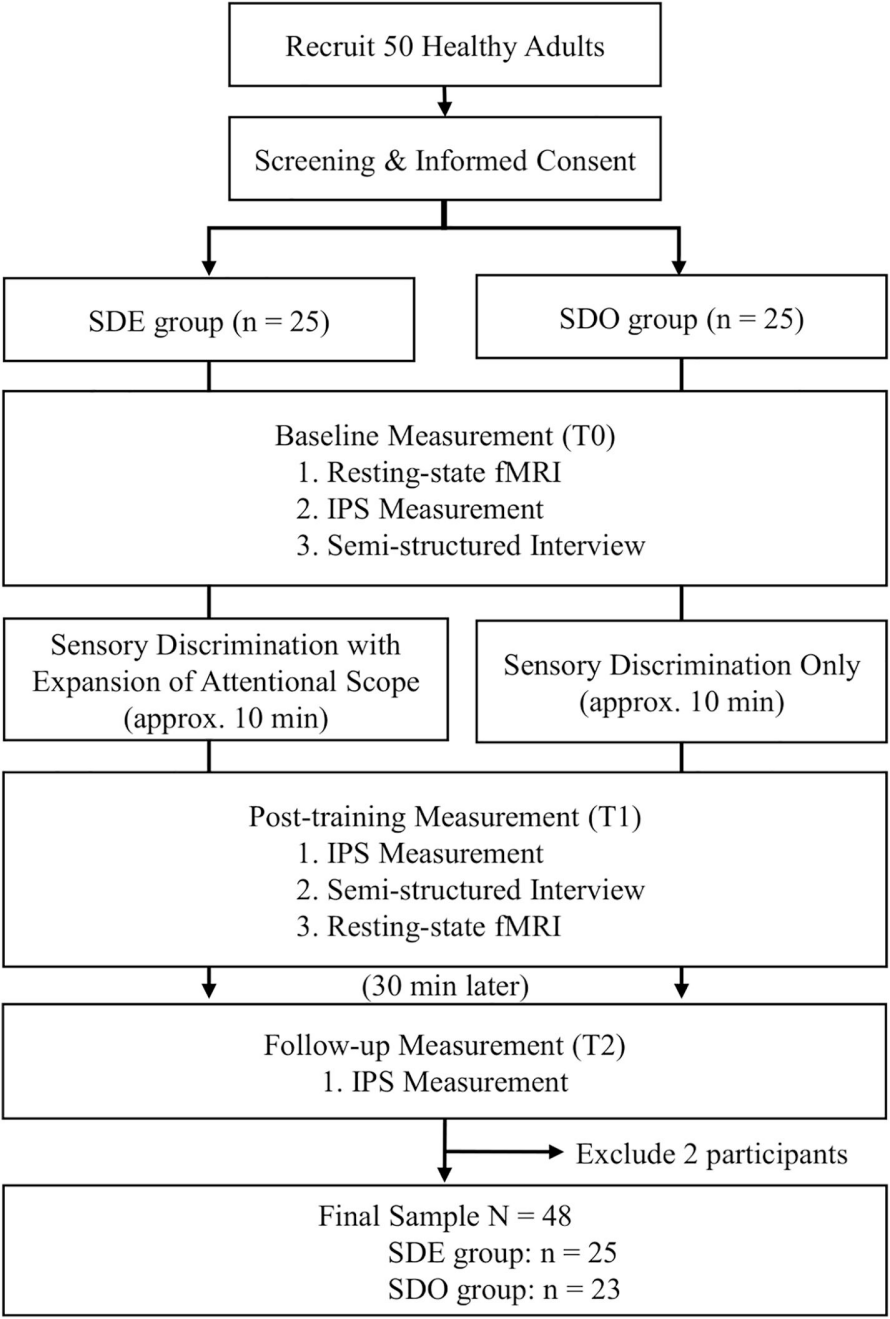
Participant flow diagram and assessment schedule. The diagram illustrates enrollment, allocation to SDE or SDO, and timing of assessments at T0 (baseline), T1 (immediately post-training), and T2 (30-min post-training); measures were collected at each time point. fMRI, functional magnetic resonance imaging; IPS, Index of Postural Stability; SDE, Sensory Discrimination with Expansion of Attentional Scope; SDO, Sensory Discrimination Only.

Participants were allocated to the SDO group (*n* = 25) or the SDE group (*n* = 25), ensuring sex balance between the groups. All 50 participants completed the experimental procedures, though two participants were excluded from the final analysis due to MRI artifacts, yielding a final sample of 48 participants (SDE group, *n* = 25; SDO group, *n* = 23) (Figure 1). The demographic characteristics of the final analytic sample after MRI-related exclusions (*N* = 48) are summarized in Table 1.

**Table 1:** Baseline demographic characteristics of participants (*N* = 48).

Characteristic	SDE group (*n* = 25)	SDO group (*n* = 23)	*p*-value	Effect size
Age (years), median [IQR]	23 [22–24.5]	23 [22–25]	.97	*r* = 0.005
Sex			.85	*φ* = 0.027
— Women, *n* (%)	19 (76)	18 (78)		
— Men, *n* (%)	6 (24)	5 (22)		
BMI, kg/m^2^, mean (SD)	21.1 (2.1)	21.1 (1.4)	.95	*r* = 0.009
EHI-LQ, median [IQR]	85 [75–95]	90 [80–100]	.32	*r* = 0.142

Demographic comparisons were performed on the final analytic sample (*N* = 48; SDE, *n* = 25; SDO, *n* = 23). Age and EHI-LQ were expressed as median [IQR] and compared between groups using the Mann–Whitney U test (effect size r). Body mass index (BMI) was expressed as the mean (SD) and compared using Welch's *t*-test (effect size, r). Sex is reported as *n* (%) and was compared using Fisher's exact test (effect size φ). Statistical significance was set at *α* = .05 (two-sided).

SDE, sensory discrimination with expansion of attentional scope; SDO, sensory discrimination only; IQR, interquartile range; SD, standard deviation; EHI-LQ, Edinburgh handedness inventory—laterality quotient.

### 2.3 Training procedure

**Figure 2:**
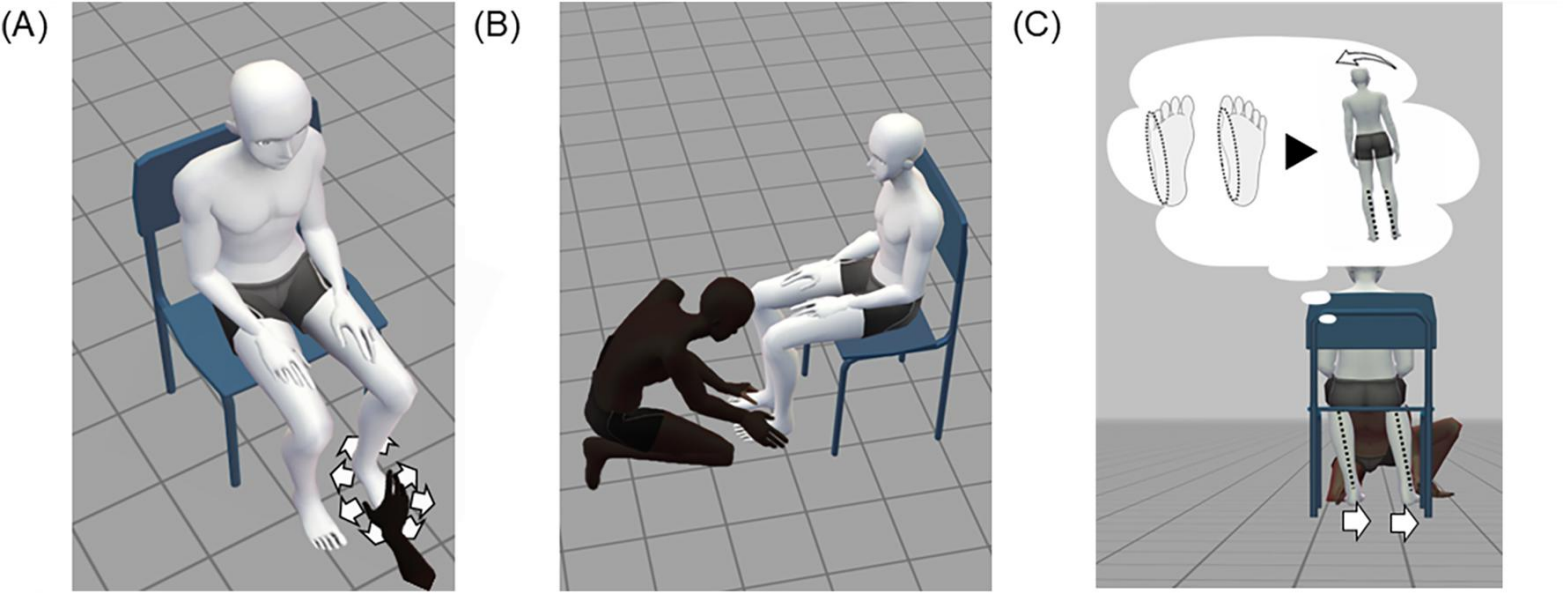
Schematic of the training procedures. All tasks were performed with feet flat on the floor. **(A)** Single-leg task (SDO/SDE, ∼5 min): The experimenter passively moved one foot ∼10 cm in one of eight directions (white arrows indicate possible directions), and the participant verbally identified the plantar region with the greatest pressure intensity. **(B)** Bilateral-leg task (SDO/SDE, ∼5 min): Both feet were passively moved simultaneously in different directions, and the participant identified the region with the greatest pressure intensity for each foot. **(C)** Attentional expansion (SDE only): During the bilateral-leg task, a brief therapeutic dialogue prompted participants to imagine their upright posture based on plantar sensation and then describe their whole-body alignment. This illustration demonstrates an example where both feet move to the right (white arrows), eliciting an imagined leftward body tilt. SDO, Sensory Discrimination Only; SDE, Sensory Discrimination with Expansion of Attentional Scope.

Participants underwent one of the following training protocols, according to their group allocation, each consisting of a single 10-minute session. All procedures were performed with participants seated on an adjustable chair, ensuring that both feet rested flat on the floor (Figure 2).

#### 2.3.1 SDO group

The SDO group training involved a plantar sensory discrimination task designed to enhance the perceptual accuracy of bottom-up sensory input from the plantar surface. This training framework was designed based on prior findings that plantar sensory discrimination can enhance somatosensory function and balance (8, 9). In this study, the experimenter passively moved each participant's foot to modify plantar pressure distribution, after which participants were asked to verbally identify the plantar region where pressure felt most intense.

The procedure comprised two parts: a single-leg task (approximately 5 min) and a bilateral-leg task (approximately 5 min). First, in the single-leg task, the experimenter repeatedly moved one foot approximately 10 cm in one of eight directions (anterior, posterior, right, left, anterior-right, anterior-left, posterior-right, or posterior-left) (Figure 2A). After each trial, participants were asked to verbally identify the plantar region where pressure felt most intense. For instance, if the foot was moved anteriorly, the biomechanically accurate response was the heel, corresponding to the anticipated shift in COP. If the answer was incorrect, the experimenter provided feedback by first moving the foot to the reported (incorrect) location, then to the correct one, to emphasize the perceptual distinction. The number of trials was varied based on the participants’ responses. During the bilateral-leg task, both feet were moved simultaneously in different directions (Figure 2B). The participants were asked to verbally identify the area of maximum plantar pressure on each foot.

#### 2.3.2 SDE group

The SDE group performed the same initial single-leg sensory discrimination task as the SDO group, but the subsequent bilateral-leg task incorporated an additional attentional expansion component. This expansion incorporated brief therapeutic dialogue, a structured conversational technique known to enhance awareness of bodily sensations and facilitate the reorganization of motor strategies (27). Through therapeutic dialogue, participants were guided to use plantar sensations as a perceptual anchor and then extend their attention from the feet to a whole-body reference frame. The conceptual foundation of this protocol was that postural control involves coordinated full-body regulation, not merely localized sensory processing (12).

For the first 5 min, the participants completed the single-leg discrimination task, identical to that in the SDO protocol. In the final 5 min, therapeutic dialogue was introduced during the bilateral-leg task phase. After each movement, once the participant identified the primary pressure region, the experimenter posed a two-step reflective prompt to promote top-down attentional expansion. First, participants were asked, “If you imagine standing straight with that foot sensation, which way would your body tilt?” This question elicited mental imagery of postural shifts. Next, they were asked, “In that posture, what is the positional relationship between the feet, knees, pelvis, and shoulders?” This question intentionally expanded the participants’ attentional scope to the entire body, facilitating the reconstruction of a global motor strategy guided by plantar sensation (Figure 2C).

### 2.4 Behavioral measures: postural stability

#### 2.4.1 IPS data acquisition

Postural stability was quantified using the Index of Postural Stability (IPS) (28) developed by Mochizuki et al. and widely used in subsequent clinical studies (29, 30). Measurements were collected at three time points: baseline (T0), immediately post-training (T1), and 30 min post-training (T2).

During each measurement, the participants stood on a stabilometer (Gravicorder GS-11; ANIMA Corp., Japan; 20 Hz) with their feet spaced 10 cm apart, and they gazed at a fixation point 2 meters ahead. Data were recorded for 10 s across five distinct positions: first, in a natural central stance, and then while participants shifted their COP to the anterior, posterior, right, and left limits of their base of support.

IPS was quantified according to the method described by Mochizuki et al. First, the rectangular area derived from the maximum anteroposterior and mediolateral diameters of the COP trajectory was calculated for each of the five measurement positions, and the average value was defined as the postural sway area. Next, the rectangular area formed by the center of sway during the four directional weight shifts (anterior, posterior, right, and left) was defined as the area of stability limit. Finally, IPS was calculated using the following formula:

$$IPS=\log\;\left(\frac{\mathrm{Area}\;\mathrm{of}\;\mathrm{Stability}\;\mathrm{Limit}+\mathrm{Area}\;\mathrm{of}\;\mathrm{Postural}\;\mathrm{Sway}}{\mathrm{Area}\;\mathrm{of}\;\mathrm{Postural}\;\mathrm{Sway}}\right)$$

Based on this formula, higher IPS values indicate greater postural stability.

#### 2.4.2 IPS statistical analysis

Statistical analysis of IPS data was performed using the R software (version 4.5.1). A 2 (group: SDO, SDE) × 3 (time: T0, T1, T2) mixed-design ANOVA (split-plot ANOVA) was performed. Generalized eta-squared (G*η*^2^) was calculated as a measure of the effect size. The Greenhouse–Geisser correction was applied if the assumption of sphericity was violated. If a significant interaction was identified, simple main effects were examined using Shaffer's method for multiple-comparison adjustment. Statistical significance was set at *α* = .05 (two-sided). To assess changes at the individual level, the difference between T1 and T0 (*Δ*IPS) was calculated and compared against the previously reported Minimal Detectable Change (MDC95) of 0.26, which served as a threshold for change beyond measurement error (31). Furthermore, within-group changes between time points were quantified using Cohen's d_x_ (within-subject effect size). Interpretation followed Cohen (1988): 0.20 (small), 0.50 (medium), and 0.80 (large) (32).

### 2.5 Qualitative measures: self-reported postural strategies

#### 2.5.1 Interview procedure

Semi-structured interviews exploring participants’ postural control strategies were performed at T0 and T1. This approach is a recognized qualitative research approach that ensures data collection consistency through a predefined interview guide while preserving flexibility to ask spontaneous follow-up questions based on participant responses (33). The primary advantage of this approach lies in its ability to delve into the underlying reasons and processes (“why” and “how”) behind the phenomena, dimensions that quantitative data alone cannot capture, adding explanatory depth (34). The interview guide focused on postural control strategies during a four-directional weight-shifting task used for IPS assessment. The main questions were: (i) “What did you pay attention to when tilting your posture forward, backward, left, and right?” and (ii) “If there were any specific body parts or sensations you were aware of, please describe how you were aware of them.” All participants received these questions in the same sequence. The same interview was performed at T1 to capture the changes immediately after training. The T1 interview was performed before any explanation, review, or debriefing of the training protocol to avoid eliciting subjective evaluations of the training itself.

All interviews were performed by the experimenter (K.G.) in a one-on-one setting in a quiet, private room and were recorded using an integrated circuit recorder. The audio data were initially transcribed using automatic speech recognition software, followed by manual proofreading by a co-author (R.S.) who was not involved in performing the interviews. Group and time-point information was not disclosed to the proofreader. The accuracy of the final transcripts was verified through random sampling and cross-checking.

#### 2.5.2 Text mining analysis

Qualitative data were analyzed using KH Coder (v3.02c) (35, 36). We screened the transcripts to retain only task-relevant utterances and excluded utterances that were unrelated or only negated/uncertain. We then used ChaSen for morphological analysis to segment the retained transcripts into words.

##### 2.5.2.1 Stage 1: grasping the overall vocabulary

**Figure 3:**
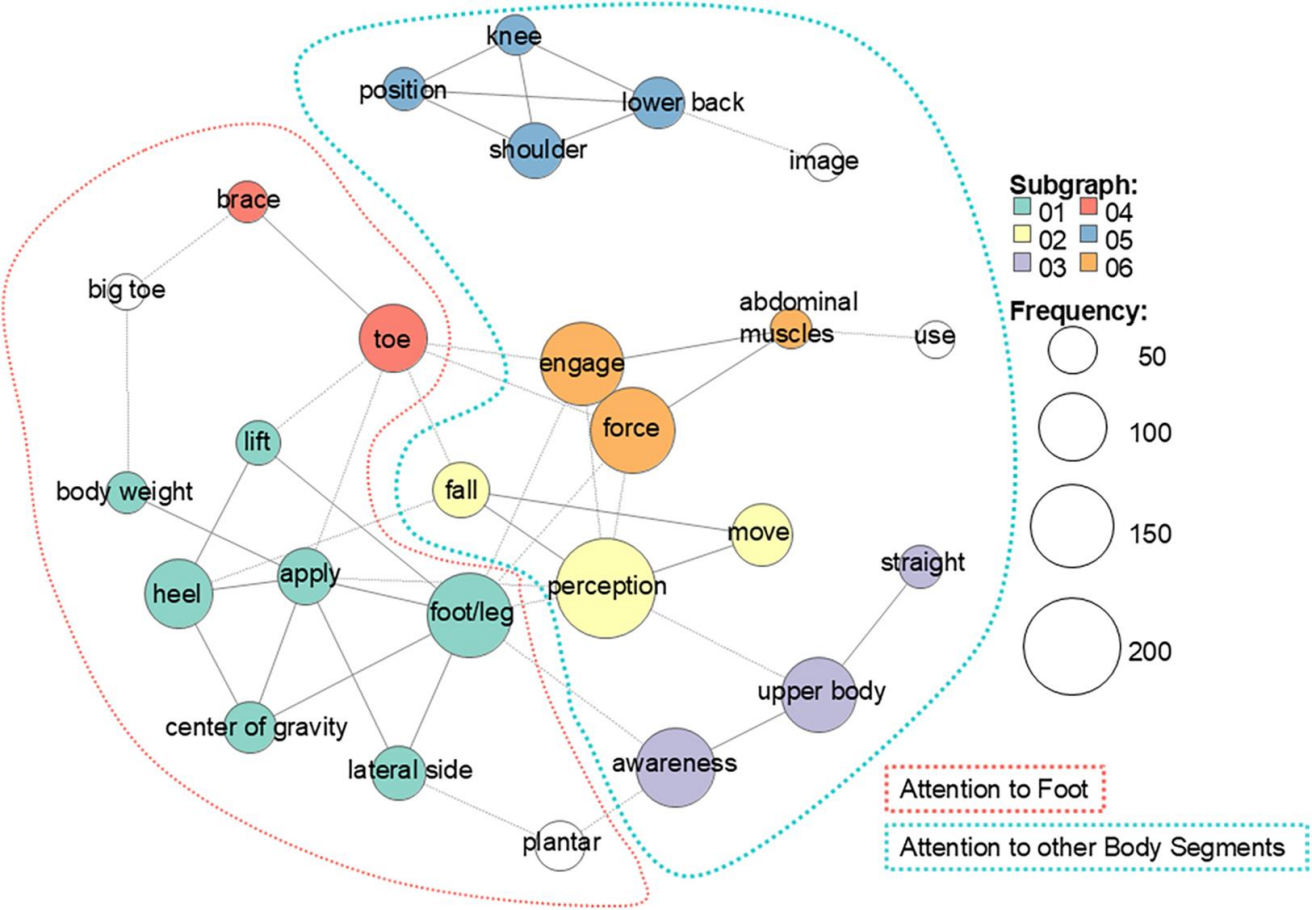
Co-occurrence network of self-reported motor-strategy vocabulary. To characterize the overall vocabulary, verbal reports from all participants at T0 and T1 were pooled into a single corpus, and words with total frequency ≥25 in the pooled corpus were analyzed. Nodes represent words (size ∝ frequency), edges link words co-occurring within the same sentence, and colors represent data-driven subgraphs. For readability, we visualized the top 45 co-occurrence edges. At a higher level, dashed contours represent two macro themes: attention to the feet (left; e.g., plantar, heel, foot/leg, and weight shift) and attention to other body segments (right; e.g., shoulder, knee, lower back, and upper body), indicating that attention was directed not only to the feet but also to other body parts.

First, to capture the overall vocabulary used in self-reported motor strategies, all verbal reports from T0 and T1 were merged into a single dataset, and a word co-occurrence network analysis was conducted. Here, a co-occurrence was defined as the simultaneous appearance of words within the same sentence. To enhance interpretability of the co-occurrence structure, we applied standard preprocessing, including a custom stop list. We also applied part-of-speech filtering and retained only content-word categories (e.g., nouns, verbs, and adjectives) for the co-occurrence analysis. To balance interpretability and representativeness, we retained only words appearing at least 25 times in the pooled corpus (all participants, T0 + T1 combined) for the co-occurrence analysis. For visualization, we plotted only the top 45 co-occurrence edges to maintain figure readability. In a sensitivity analysis retaining spatial-direction terms (e.g., “front,” “back,” “left,” “right”), these terms strongly co-occurred with foot-related terms (e.g., “foot/feet”), yielding an additional direction/trajectory-centered cluster. Because this cluster reflects directional control rather than anatomical referencing, we excluded spatial-direction terms from the primary co-occurrence network analysis and present the retained-term network in Supplementary Figure S1. The resulting co-occurrence network diagram was jointly interpreted by multiple analysts to identify the themes formed by groups of strongly related words. This analysis demonstrated that the participants’ overall vocabulary, including at T0 and T1, directed attention not only to the feet but also to various whole-body parts, such as the shoulders and trunk (Figure 3).

##### 2.5.2.2 Stage 2: coding of body part categories and group comparison.

Building on Stage 1, we conducted a coding-based analysis to quantify body-part references in participants’ self-reported motor strategies at both the participant and sentence levels, and we compared these distributions across groups and time points. Specifically, we defined five body-part codes [Foot; Lower limb (non-foot); Shoulder; Lumbopelvic region; and Trunk] with explicit coding rules and related synonyms and linguistic variants (Table 2). Two researchers experienced in text mining independently reviewed the coding rules, and final decisions were reached by consensus. Each participant's transcript at a given time point was treated as a single document. For each of the five body part codes, we assessed presence or absence within the document. If a word corresponding to a code appeared at least once, the participant's document was coded as “1” for that category; otherwise, it was coded as “0.” This process generated a binary dataset in which multiple codes could be simultaneously positive for a single participant.

**Table 2:** Coding rules for body part codes.

Code	Included terms
Foot	Plantar, sole, arch, toe, “big toe,” “ball of foot,” “little toe,” heel, foot
Lower limb (non-foot)	Knee, thigh, calf, “lower leg,” “lower body,” “hip joint"
Shoulder	Shoulder, “right shoulder,” “left shoulder"
Lumbopelvic region	“Lower back,” lumbar, pelvis, sacrum, buttocks, gluteal
Trunk	“Upper body,” navel, trunk, core, spine, body, back, chest, abdomen

Text mining analysis was performed on the original Japanese transcripts. The terms listed are English translations of the Japanese expressions used in coding.

Subsequently, we cross-tabulated these binary data by group (SDO vs. SDE) and time point (T0 vs. T1) and applied Pearson's chi-square tests to assess associations, with Cramér's V computed to quantify effect size. When the chi-square test was significant, we examined adjusted standardized residuals (|z| > 1.96) to identify codes that were over- or under-represented across groups and time points. As a sensitivity analysis to complement the primary participant-level coding, we summarized sentence-level code frequency (the number and percentage of sentences containing each code) by condition and time point and reported these descriptive results in Supplementary Table S1. All analyses were performed in Japanese. For publication, a bilingual coauthor translated the resulting co-occurrence network diagram and coding rules into English.

### 2.6 Neuroimaging measures: rs-fMRI

#### 2.6.1 MRI data acquisition

All images were captured on a 3.0T MRI scanner (Philips Achieva 3.0T) equipped with an 8-channel head coil (SENSE-Head-8). Both structural and resting-state functional images were captured during two sessions: T0 (baseline) and T1 (post-training). Within each session, data were collected in the following sequence: T0, rs-fMRI, IPS, and interview; and T1, IPS, interview, and rs-fMRI (Figure 1). At T1, behavioral assessments were performed prior to imaging to capture the immediate post-training effect. The order was identical across all groups. Participants’ head movements were minimized using foam cushions and sound-attenuating headphones.

Structural T1-weighted images were captured using a 3D Turbo Field Echo sequence with these parameters: repetition time (TR) = 8.1 ms; echo time (TE) = 3.7 ms; flip angle = 8°; field of view (FOV) = 240 × 240 mm; matrix size = 240 × 240; 160 sagittal slices; slice thickness = 1.0 mm; voxel size = 1.0 × 1.0 × 1.0 mm.

rs-fMRI images were captured using a single-shot echo-planar imaging sequence. The participants were instructed to keep their eyes open and maintain visual fixation on a crosshair presented on a screen. The main acquisition parameters were TR = 2,500 ms; TE = 30 ms; flip angle = 80°; FOV = 212 × 212 mm; matrix size = 64 × 64; 40 ascending slices; slice thickness = 3.2 mm; slice gap = 0.8 mm; voxel size = 3.31 × 3.37 × 3.20 mm. In total, 240 volumes were captured, with the first four volumes discarded to allow for magnetization stabilization. Fat suppression was performed using the Spectral Presaturation with Inversion Recovery technique.

#### 2.6.2 MRI data preprocessing

Pre-processing and subsequent statistical analysis of the rs-fMRI data were performed using the CONN toolbox (v.22a) (37), running on SPM12 (Wellcome Department of Imaging Neuroscience, London, UK).

The preprocessing pipeline followed the default CONN protocol and consisted of the following sequential steps: (1) slice-timing correction; (2) realignment and unwarping for head-motion correction; (3) outlier identification using the Artifact Detection Tools (ART); (4) coregistration of each participant's T1-weighted anatomical image; (5) tissue segmentation into gray matter, white matter (WM), and cerebrospinal fluid (CSF); (6) spatial normalization to Montreal Neurological Institute (MNI) space; and (7) spatial smoothing using a 6 mm full-width at half-maximum Gaussian kernel.

Subsequent denoising was performed using the anatomical component-based noise correction method (aCompCor). The five leading principal components of both WM and CSF masks were regressed out from the blood oxygenation level dependent (BOLD) signal. Additional nuisance regressors included 24 motion-related parameters (6 rigid-body parameters, their first-order temporal derivatives, and the squares of all 12 values). Finally, the data were linearly detrended and bandpass filtered (0.008–0.09 Hz).

#### 2.6.3 Region-of-interest (ROI)-to-ROI functional connectivity analysis

Based on the theoretical framework of the training protocol, four major large-scale networks were selected for the ROI-to-ROI analysis: the SN, the FPN, the DMN, and the SMN. These four networks comprised 18 anatomically defined ROIs derived from the CONN default atlas (SN: seven ROIs; FPN: four ROIs; DMN: four ROIs; SMN: three ROIs). The SN was included because of its central role in detecting and orienting attention toward salient stimuli (38), a process fundamental to somatosensory-focused interventions. The FPN was selected for its central role in top-down cognitive modulation required for the attentional guidance component of the SDE protocol (39). The DMN was included because of its critical role in motor readiness (40). Finally, the SMN was selected for its critical role in sensory processing and motor execution, the ultimate target of the postural control training (41).

For the first-level analysis, the mean BOLD time series was extracted for each of the 18 ROIs. For each participant, a connectivity matrix was computed by calculating Fisher's z-transformed correlation coefficients between all the ROI pairs, yielding 153 pairwise connections. The change in FC (*Δ*FC) was calculated as the difference between post- and pre-training z-values (T1—T0).

For the second-level analysis, a general linear model was employed to assess the interaction effect of Group (SDE vs. SDO) × Time (T1 vs. T0) on FC. To correct for multiple comparisons across 153 connections, a nonparametric permutation-based Threshold-Free Cluster Enhancement (TFCE) approach was employed (42). Statistical significance was defined as a cluster-level family-wise error (FWE)-corrected *p*-value of *p* < .05. For any significant cluster identified, the t-values and false discovery rate (FDR)-corrected *p*-values of individual ROI-to-ROI connections were extracted for *post-hoc* interpretation.

#### 2.6.4 Exploratory association with behavioral change

To explore the relationship between the observed changes in brain connectivity and behavior, we performed an exploratory correlation analysis. First, for the significant cluster identified in the primary analysis, the mean change in FC (*Δ*FC_cluster) was calculated across all its constituent connections for each participant. Spearman's rank correlation was then employed to examine the association between *Δ*FC_cluster and the improvement in postural stability (*Δ*IPS) separately for each group.

In a complementary *post hoc* analysis, we further examined the relationship between *Δ*IPS and edge-specific FC changes in the single connection that remained significant after FDR correction (right AInsula–right SMG; *Δ*FC_edge). This correlation was assessed using Spearman's rank correlation for each group. As an exploratory complement to these change-score analyses, we examined time point–specific (concurrent) correlations between IPS and FC at T0 and T1 for both the cluster-level metric (FC_cluster) and the significant edge (FC_edge), separately by group (Supplementary Figure S2).

## 3 Results

### 3.1 Participant characteristics

A total of 48 participants were included in the final analysis (SDE group: *n* = 25; SDO group: *n* = 23). As presented in Table 1, there were no significant baseline differences between the two groups in terms of age, sex, body mass index (BMI), or handedness score (all *p* > .05).

### 3.2 Behavioral results: postural stability (IPS)

**Figure 4:**
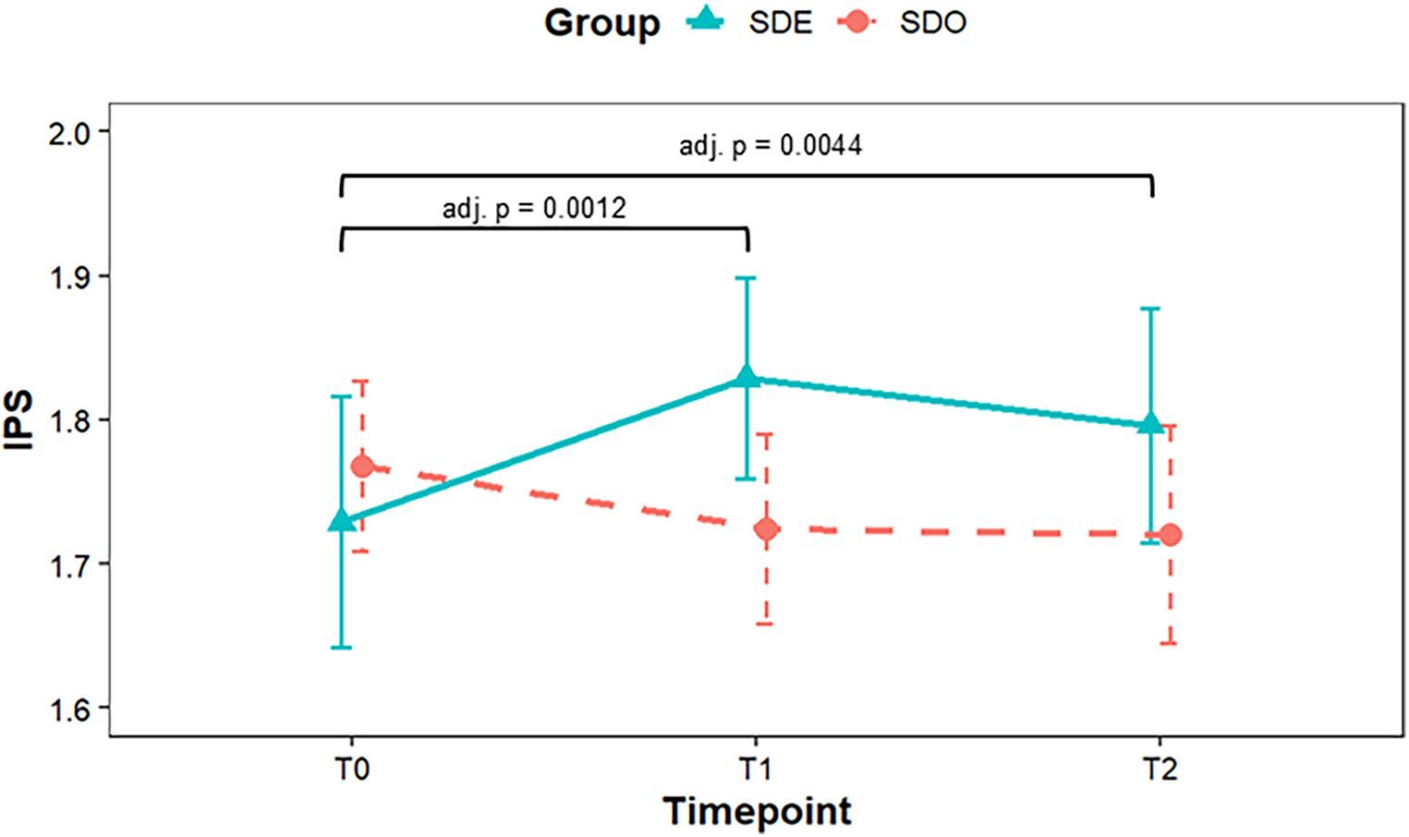
Changes in the Index of postural stability (IPS) over time by group. Points represent group means (SDE, solid; SDO, dashed) with 95% CIs as error bars; lines connect means across time points (T0 baseline, T1 immediately post-training, T2 30-min post-training; higher values indicate better stability). Split-plot ANOVA demonstrated a significant Group × Time interaction. In SDE, IPS increased from T0 to T1 and remained higher at T2 (brackets denote within-group pairwise tests with Shaffer-adjusted p-values), whereas SDO showed no apparent change. ANOVA, analysis of variance; CI, confidence interval; IPS, Index of Postural Stability; SDE, Sensory Discrimination with Expansion of Attentional Scope; SDO, Sensory Discrimination Only; T0, baseline; T1, immediately post-training; T2, 30-minute post-training.

A mixed-design (split-plot) ANOVA was performed with factors Group (SDO, SDE) and Time (T0–T2) for IPS. The analysis revealed a significant Group×Time interaction, *F*(1.80, 82.90) = 5.83, *p* = .004, G*η*^2^ = .017 (Greenhouse–Geisser corrected), indicating that the temporal pattern of postural stability changes differed between the groups. Following this interaction, Shaffer's method was used to test the simple main effects. The SDE group demonstrated a significant increase in IPS from T0 to T1 (*Δ*IPS = + 0.09, *p* = .001, dz = 0.42), and this improvement persisted at T2 (T0 vs. T2: *p* = .004, dz = 0.32). In contrast, the SDO group demonstrated no significant within-group changes. Although the mean IPS change in the SDE group did not exceed the previously reported MDC95 of 0.26 for young healthy adults, the improvement was statistically significant. The time course is illustrated in Figure 4, and the descriptive statistics and test values are summarized in Table 3.

**Table 3:** Descriptive statistics and within-group changes in index of postural stability.

Time point/statistics	SDE group (*n* = 25)	SDO group (*n* = 23)
IPS mean (SD)		
T0 (Baseline)	1.71 (0.23)	1.75 (0.13)
T1 (Post)	1.80 (0.22)	1.73 (0.16)
T2 (Follow-up)	1.78 (0.24)	1.73 (0.18)
Within-Group Change *Δ*(T1–T0)		
Mean change [95% CI]	+0.09 [0.05, 0.14]	−0.02 [−0.07, 0.02]
adjusted p (SDE only); Cohen's dz	.001; 0.42	n/a; −0.15
Within-Group Change *Δ*(T2–T0)		
Mean change [95% CI]	+0.07 [0.03, 0.12]	−0.02 [−0.08, 0.04]
adjusted p (SDE only); Cohen's dz	.004; 0.32	n/a; −0.15
Within-Group Change *Δ*(T2–T1)		
Mean change [95% CI]	−0.02 [−0.07, 0.03]	−0.00 [−0.08, 0.07]
adjusted p (SDE only); Cohen's dz	.425; −0.08	n/a; −0.01

Values are expressed as mean (SD) unless otherwise noted. *Δ* denotes the difference between later and earlier time points. Brackets denote 95% CIs for the mean change. Following a significant Group × Time interaction in the main analysis, within-group Shaffer-adjusted *p*-values are reported for the SDE group. dz is Cohen's paired standardized mean change calculated from the SD of differences and is reported for both groups to indicate magnitude, irrespective of significance. Two-sided *α* = .05.

CI, confidence interval; IPS, index of postural stability; SDE, sensory discrimination with expansion of attentional scope; SDO, sensory discrimination only; SD, standard deviation.

### 3.3 Qualitative results: self-reported motor strategies

As demonstrated in Table 4, the distribution of body part codes derived from participants’ self-reported motor strategies demonstrated a statistically significant association with group and time point [overall *χ*^2^(12) = 30.41, *p* = .002, Cramér's V = 0.20]. Expected counts were calculated under the null hypothesis of independence based on the marginal totals. In the SDE group at T1, the observed count for the “Shoulder” code was higher than the expected count (z = 4.83, *p* < .001), whereas the “Foot” code was lower than the expected count (z = −2.37, *p* = .018). At T0 in the SDE group, the observed count for the “Shoulder” code was lower than the expected count (z = −3.01, *p* = .003). No significant deviations were observed for any other body part codes, nor in the SDO group at either time point (all |z| < 1.96). As a sensitivity analysis, sentence-level code frequency is summarized descriptively in Supplementary Table S1 to complement the participant-level findings. Across conditions, the Foot code remained prevalent, whereas the primary qualitative change in SDE at T1 was the marked increase in Shoulder references, consistent with attentional expansion rather than a shift away from foot-related monitoring.

**Table 4:** Frequencies and adjusted standardized residuals for body part codes by group and time point.

Code	SDE at T0	SDE at T1	SDO at T0	SDO at T1
	*n* (%), z	*n* (%), z	*n* (%), z	*n* (%), z
Foot	25 (100%), 0.79	24 (96.0%), −2.37^a^	22 (95.7%), 1.02	20 (87.0%), 0.98
Lower limb (non-foot)	7 (28.0%), 0.17	12 (48.0%), 0.97	3 (13.0%), −1.30	5 (21.7%), −0.03
Shoulder	0 (0.0%), −3.01^a^	20 (80.0%), 4.83^a^	2 (8.7%), −1.65	3 (13.0%), −0.91
Lumbopelvic region	9 (36.0%), 0.16	14 (56.0%), 0.54	7 (30.4%), −0.11	5 (21.7%), −0.73
Trunk	22 (88.0%), 0.88	22 (88.0%), −1.71	19 (82.6%), 0.96	15 (65.2%), 0.13

The percentages are within-group denominators (SDE, *n* = 25; SDO, *n* = 23). The entries demonstrate participant-level presence/absence coding: the number of participants in whom each code appeared at least once (n), the corresponding percentage (%), and the adjusted standardized residual (z). Expected counts were derived from the marginal totals under the null hypothesis of independence.

SDE, sensory discrimination with expansion of attentional scope; SDO, sensory discrimination only.

^a^ Indicates |z|≥1.96. Multiple comparison adjustments were not applied to the residuals. To complement this participant-level analysis, a sentence-level sensitivity analysis is provided in Supplementary Table S1.

### 3.4 Neuroimaging results: rs-fMRI (ROI-to-ROI, cluster-level)

**Figure 5:**
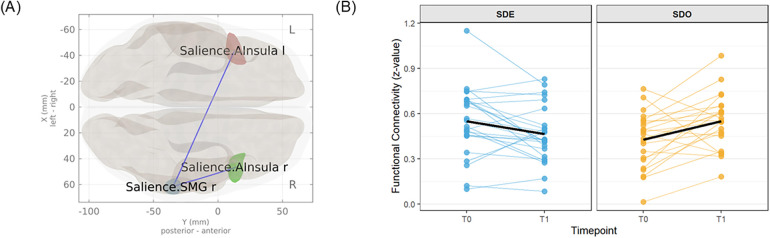
Training-related changes in resting-state functional connectivity. **(A)** The two edges comprise the single TFCE-significant cluster for the Group × Time interaction (cluster-level p-FWE <.05); see Table 5 for edgewise statistics. **(B)** The slope plot demonstrates edge-averaged (cluster-mean) Fisher z-transformed connectivity from T0 to T1 for each individual (thin lines) and the group mean (bold line). Cluster-mean connectivity was computed as the mean of the two constituent ROI-to-ROI Fisher's z values at each time point. On average, mean connectivity within the TFCE-significant cluster (i.e., the two constituent ROI-to-ROI connections) decreased in SDE and increased in SDO. AInsula, anterior insula; l/r, left/right; SMG, supramarginal gyrus; SDE, Sensory Discrimination with Expansion of Attentional Scope; SDO, Sensory Discrimination Only; T0, baseline; T1, immediately post-training; TFCE, threshold-free cluster enhancement; p-FWE, family-wise error-corrected p-value.

The ROI-to-ROI analysis assessed the Group × Time interaction on *Δ*FC (T1—T0). One significant cluster was identified within the ROIs defined for the SN, TFCE = 36.29, p-FWE = .015 (Figure 5; Table 5). No significant clusters were identified in the other predefined networks. The mean connectivity within this two-edge SN cluster decreased following training in the SDE group, whereas it tended to increase in the SDO group (Figure 5B). As demonstrated in Table 5, this cluster comprised two connections. Negative t-values indicated that the change in connectivity (T1—T0) in the SDE group was significantly smaller than that in the SDO group. *post-hoc* inspection of the individual connections within this cluster demonstrated that the change in connectivity between the right anterior insula (rAI) and the right supramarginal gyrus (rSMG) remained significant following FDR correction (p-FDR = .012). In contrast, the difference between the left anterior insula and rSMG did not reach significance (p-FDR = .336).

**Table 5:** Attentional-circuitry edges (salience/ventral attention) comprising the TFCE-significant cluster for the group × time interaction (ROI-to-ROI FC).

Cluster	Brain region 1, MNI coordinates (x, y, z)	Brain region 2, MNI coordinates (x, y, z)	T-value (df = 46)	p-FDR
Cluster 1 (TFCE = 36.29, p-FWE = 0.015)	rAInsula, (47, 14, 0)	rSMG, (62, −35, 32)	−4.35	0.012
	lAInsula, (−44, 13, 1)	rSMG, (62, −35, 32)	−2.71	0.336

The general linear model tested the Group [sensory discrimination with expansion of attentional scope [SDE] and sensory discrimination only [SDO]] × Time (T1 vs. T0) interaction on Fisher's z-transformed ROI-to-ROI functional connectivity (FC). The subheader presents cluster-level statistics derived from threshold-free cluster enhancement (TFCE) with family-wise error correction (p-FWE). For *post hoc* inspection within the significant cluster, edgewise *p*-values were FDR-corrected; only the right AInsula–right SMG edge remained significant (p-FDR = .012). Negative *t* values indicate a greater post-pre decrease in FC in SDE than in SDO (i.e., *Δ*FC_SDE < *Δ*FC_SDO). MNI coordinates (mm) are ROI centroids from the CONN atlas.

AInsula, anterior insula; FC, resting-state functional connectivity; FDR, false discovery rate; FWE, family-wise error; l/r, left/right; MNI, Montreal Neurological Institute; SMG, supramarginal gyrus; SN, salience network; VAN, ventral attention network.

### 3.5 Exploratory association with IPS

**Figure 6:**
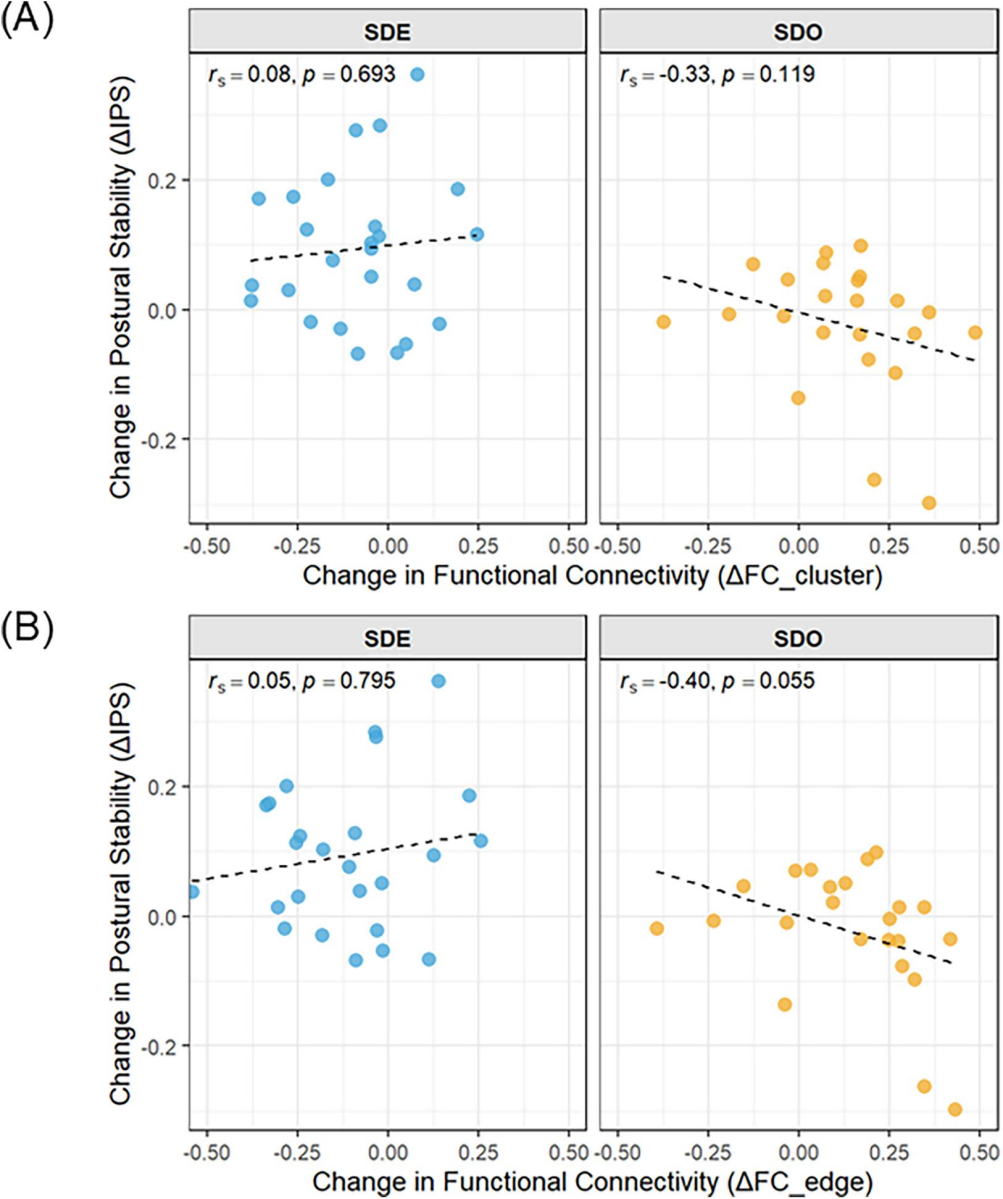
Exploratory correlations between changes in resting-state functional connectivity and postural stability. Scatter plots demonstrate the correlation between changes in FC (*Δ*FC; *x*-axis) and IPS (*Δ*IPS; *y*-axis) by group. **(A)** *Δ*FC of the TFCE-significant cluster (*Δ*FC_cluster). **(B)** *Δ*FC of the edge that survived FDR correction (p-FDR <.05; right AInsula–right SMG; *Δ*FC_edge). Points represent individuals, dashed lines indicate least-squares fits, and Spearman's rs and *p*-values are annotated. Correlations in the SDO group were of moderate negative magnitude (panel A: rs = −0.33; panel B: rs = −0.40), though they did not reach statistical significance (*p* = .119 and *p* = .055, respectively). Correlations in the SDE group were negligible and non-significant. *Δ*, change (T1 minus T0); FC, resting-state functional connectivity; FDR, false discovery rate; IPS, index of postural stability; SDE, sensory discrimination with expansion of attentional scope; SDO, sensory discrimination only; rs, spearman's rank correlation coefficient.

For the significant cluster, we assessed the relationship between its mean FC change (*Δ*FC_cluster) and *Δ*IPS using Spearman's rank correlation. In the SDO group, the correlation with *Δ*IPS was of moderate negative magnitude (rs = −0.33), but it did not reach statistical significance (*p* = .119; Figure 6A). In contrast, in the SDE group, the correlation with *Δ*IPS was close to zero (rs = 0.08, *p* = .693).

In an additional *post-hoc* exploratory analysis, we also assessed the relationship between *Δ*IPS and the change in the single edge that remained significant following FDR correction (rAI–rSMG; *Δ*FC_edge). In the SDO group, this connection showed a correlation with *Δ*IPS that was of moderate negative magnitude (rs = −0.40), though this association also did not reach statistical significance (*p* = .055; Figure 6B). In contrast, the correlation in the SDE group was negligible (rs = 0.05, *p* = .795).

## 4 Discussion

This study found that a brief training protocol designed to expand the attentional scope from localized plantar sensations used as a perceptual anchor toward a whole-body reference frame was associated with immediate, multilevel effects, encompassing behavioral, subjective, and neurophysiological measures. Consistent with our hypotheses, convergent evidence was observed across these three levels. A significant Group × Time interaction was observed for postural stability. The SDE group demonstrated an increase in the IPS immediately post-training, which persisted 30 min later, whereas the SDO group demonstrated no change. Although the mean *Δ*IPS did not exceed the MDC95 threshold, a statistically robust improvement was nevertheless observed at the group level. Complementing this behavioral effect, analysis of self-reported motor strategies suggested a marked post-training increase in the “Shoulder” code in the SDE group, alongside a modest reduction in the “Foot” code at the participant level. This pattern is consistent with attentional expansion toward whole-body strategy elements while foot-related monitoring remained common, rather than a complete shift away from foot-focused attention (Table 4). Furthermore, a significant Group × Time interaction was identified in resting-state FC in one connection (rAI–rSMG), in which connectivity decreased in the SDE group, whereas connectivity demonstrated a rising trend in the SDO group. Collectively, these convergent results suggest that attentional-scope expansion was associated with immediate improvements in postural stability and with concurrent changes in self-reported motor strategies and post-training resting-state connectivity.

The core mechanism of this training may involve reconfiguring how bottom-up sensory information is utilized. It is proposed that using plantar sensations as a perceptual anchor to expand the attentional scope toward a whole-body reference frame may promote attentional expansion toward more global motor strategy elements, which could contribute to immediate postural improvements. Importantly, this expansion does not imply disengaging from plantar monitoring; rather, plantar sensations remain a perceptual anchor while attention broadens to include additional whole-body reference cues. Prior studies have demonstrated that interventions promoting top-down attentional control of the whole body, such as Body Awareness Therapy (43) and body scan techniques in mindfulness meditation (44), can improve balance and interoceptive accuracy. The improvements observed, coupled with an expansion of attention to include whole-body reference cues, are consistent with these findings. However, the unique aspect of our approach lies in integrating conventional sensory refinement techniques (8, 9) with explicit therapeutic dialogue. This dialogue guides participants to use localized plantar sensations as a perceptual anchor and then broaden their attention to a whole-body reference frame, thereby promoting the learning of inter-segment coordination. This integrative process may facilitate active coordination between bottom-up sensory information and top-down attentional control, potentially supporting postural control. This integrative framework may offer a novel perspective for addressing the limitations of conventional sensory-focused rehabilitation and may inform future work on sensory input augmentation and discrimination training.

A further notable finding was the opposing direction of FC change between the rAI and the rSMG. This divergence likely reflects the distinct attentional demands elicited by each training protocol. The rAI, a central hub of the SN, plays a pivotal role in initiating attentional control in response to behaviorally salient stimuli (45). In contrast, the rSMG, which is included among the SN ROIs in the CONN atlas, is functionally known to be part of the right temporoparietal junction (rTPJ), a key hub of the Ventral Attention Network (VAN) (46). The VAN primarily supports the bottom-up reorientation of attention toward behaviorally relevant external stimuli. The SDO protocol, emphasizing discrimination of sequential, externally guided foot movements, inherently prioritizes the reactive and localized modes of attention. Its structure, which included a sequence of discrete movements, required participants to repeatedly shift their focus to each new transient plantar sensation. This process of continuous, reactive monitoring of externally generated stimuli may require relatively strong functional coupling between the SN (detecting the new sensation) and the VAN (shifting attention to it). The observed increase in rAI–rSMG connectivity in the SDO group may therefore be consistent with a cognitively demanding, stimulus-driven mode of attention in which participants repeatedly monitor and shift to each new plantar sensation. However, exploratory correlations between changes in this connectivity and postural improvement in the SDO group were of moderate negative magnitude (rs = –0.40), although they did not reach statistical significance (*p* = .055). Accordingly, these correlations cannot be taken as evidence for or against this interpretation. At present, the idea that heightened SN–VAN integration reflects a relatively inefficient configuration for enhancing stability should be regarded as a speculative hypothesis that requires testing in future studies. In contrast, the SDE protocol may have reframed the role of stimulus-driven inputs. Rather than treating plantar sensation as an isolated target for reactive monitoring, it was designed to guide participants to use this sensory signal as a perceptual anchor to facilitate a shift in top-down attentional control from a localized focus to a more global awareness of whole-body alignment. Encouraging participants to consciously link plantar sensory information to their overall body alignment may have cultivated a more efficient attentional state that may rely less on continuous, tightly coupled SN–VAN interaction. One potential explanation is that expanding the attentional scope could recast the plantar sensory input as a whole-body reference signal, thereby potentially contributing to changes in SN–VAN coupling. Consistent with this interpretation, these connectivity changes were accompanied by short-term gains in postural stability, although the present data do not establish a mechanistic link between the neural and behavioral effects.

These seemingly opposing patterns of changes in neural activity between the two groups may be discussed within the higher-order framework of functional segregation and integration. According to Wang et al., efficient brain function relies on a dynamic equilibrium between segregation, in which individual networks perform independently for specialized processing, and integration, in which multiple networks coordinate to handle complex tasks (47). Conceptually, when integration becomes excessively strong or widespread, neural processing may paradoxically lose efficiency, the distinct roles of specialized networks blur, and overall cost-effectiveness diminishes (48). In light of this context, the increase in rAI–rSMG connectivity observed in the SDO condition from T0 to T1 may be consistent with a more strongly coupled state, whereas the decrease in rAI–rSMG connectivity in the SDE condition may be consistent with relatively greater segregation. However, because we did not observe a significant *Δ*FC–*Δ*IPS association, these interpretations remain speculative and should be regarded as associative rather than mechanistically established. Accordingly, attentional expansion may have accompanied both strategy-level changes and short-lived network-state differences without demonstrating a causal neural mechanism.

This study has some limitations. First, the same physical therapist administered all training, IPS assessments, and interviews, and neither allocation concealment nor masking was applied. These factors may have introduced expectancy effects, observer bias, and interviewer bias, potentially inflating apparent improvements in the SDE group. Although the T1 interviews were conducted before any debriefing and instructions were standardized, residual bias cannot be excluded. Future studies should employ independent, blinded assessors for IPS testing and interviews, and should mask analysts to group allocation when feasible. Second, although the protocol aimed to use plantar cutaneous sensation as a perceptual anchor, participants could also have relied on other inputs (e.g., ankle/knee proprioception or vision) to judge posture and respond during training. In addition, because therapeutic dialogue was an integral component of SDE, some post-training vocabulary may have been seeded by the dialogue. Conversely, low-frequency but potentially meaningful expressions may be under-represented by frequency-based text-mining outputs, warranting complementary qualitative analyses in future work. Third, although the SDE group showed a statistically reliable improvement in IPS, the mean change (*Δ*IPS ≈ + 0.09) did not exceed the minimal detectable change threshold (MDC95 = 0.26) reported for young healthy adults. Therefore, the observed group-level effect may not indicate a meaningful change for an individual based on this MDC criterion. Fourth, this proof-of-concept study was conducted in a small, homogeneous sample of young, healthy adults and used a single brief session; therefore, generalizability to clinical populations and the durability of effects remain unknown. Fifth, rs-fMRI data were collected immediately before and after training, and the exploratory analyses did not identify a significant brain–behavior association; accordingly, the relationship between the observed connectivity change and postural improvement remains associative rather than mechanistically established. This null association should be interpreted cautiously, because correlations based on change scores can be particularly sensitive to measurement error (49). Future studies employing multi-session designs with follow-ups over days to weeks are necessary to determine whether these network changes persist and to clarify their mechanistic association with behavioral outcomes. Overall, these findings should be interpreted as mechanistic, multi-level proof-of-concept evidence of immediate post-training changes that can guide hypotheses for future mechanistic and longitudinal studies, rather than as evidence of clinical effectiveness.

## 5 Conclusion

This proof-of-concept study in healthy young adults suggests that expanding the attentional scope from localized plantar sensations, used as a perceptual anchor, toward a whole-body reference frame was associated with immediate changes in postural stability, self-reported motor strategies, and resting-state connectivity. Enhancing sensitivity to bottom-up plantar sensory input remains a fundamental and important goal for postural control. Nevertheless, our results suggest a potentially important next step: learning how to interpret and use plantar input not as an isolated stimulus, but as a whole-body reference signal for balance regulation. At the same time, the single-session design and the absence of a significant brain–behavior association limit mechanistic inference and conclusions about durability. Further mechanistic and longitudinal studies, including evaluation in clinical populations, are warranted.

## Data Availability

The raw data supporting the conclusions of this article will be made available by the authors, without undue reservation.

## Supplementary material

Supplementary Figure S1 Co-occurrence network of self-reported motor-strategy vocabulary when spatial-direction terms are retained. To illustrate the impact of retaining spatial-direction terms, verbal reports from all participants at T0 and T1 were pooled into a single corpus, and words with total frequency ≥25 in the pooled corpus were analyzed. Nodes represent words (size ∝ frequency), edges link words co-occurring within the same sentence, and colors represent data-driven subgraphs. For readability, we visualized the top 45 co-occurrence edges. When spatial-direction terms (e.g., “front,” “back/backward,” “left,” “right”) are retained, they co-occur strongly with foot-related terms and form a prominent direction/trajectory-centered cluster (dashed outline: Cluster dominated by spatial-direction terms), which can dominate the network structure under top-edge visualization and reduce interpretability for anatomical referencing. This figure is provided to justify excluding spatial-direction terms from the primary co-occurrence network (Figure 3). Abbreviations: T0, baseline; T1, immediately post-training

Supplementary Figure S2 Exploratory cross-sectional correlations between resting-state functional connectivity and postural stability at baseline and post-training. Scatter plots demonstrate the correlation between resting-state functional connectivity (Fisher's z; *x*-axis) and postural stability (IPS; *y*-axis) at T0 (baseline) and T1 (post-training), plotted by group. **(A)** Cluster-level FC (FC_cluster) of the TFCE-significant cluster. **(B)** Edge-level FC (FC_edge) of the connection that survived FDR correction (p-FDR < .05; right AInsula–right SMG). Points represent individuals, dashed lines indicate least-squares fits, and Spearman's r_s_ and *p*-values are annotated. These analyses were conducted as exploratory, cross-sectional complements to the primary change-score correlations. FC, resting-state functional connectivity; IPS, index of postural stability; FDR, false discovery rate; SDE, sensory discrimination with expansion of attentional scope; SDO, sensory discrimination only; r_s_, spearman's rank correlation coefficient; rAI, right anterior insula; SMG, supramarginal gyrus; TFCE, threshold-free cluster enhancement; T0, baseline; T1, post-training.

Supplementary Table S1 Sentence-level frequencies for body-part codes by group and time point (sensitivity analysis). Percentages are calculated using the total number of sentences within each group × time point as the denominator (SDE at T0, *n* = 260; SDE at T1, *n* = 277; SDO at T0, *n* = 197; SDO at T1, *n* = 189). Entries show the number of sentences containing each code (n) and the corresponding percentage (%). Because a single sentence could contain multiple body-part codes (multi-label), column totals may not sum to the total number of sentences. This table is provided as a descriptive sensitivity analysis to complement the primary participant-level (presence/absence) coding (Table 4). SDE, sensory discrimination with expansion of attentional scope; SDO, sensory discrimination only.
