# Supplementary figures and images for: Expansion of attentional scope modulates postural control, motor strategies, and attentional network connectivity in healthy adults: a proof-of-concept mixed-methods study
Source: Front Rehabil Sci. 2026 Mar 12;7:1758682. doi: 10.3389/fresc.2026.1758682 (PMC13018112; doi:10.3389/fresc.2026.1758682)

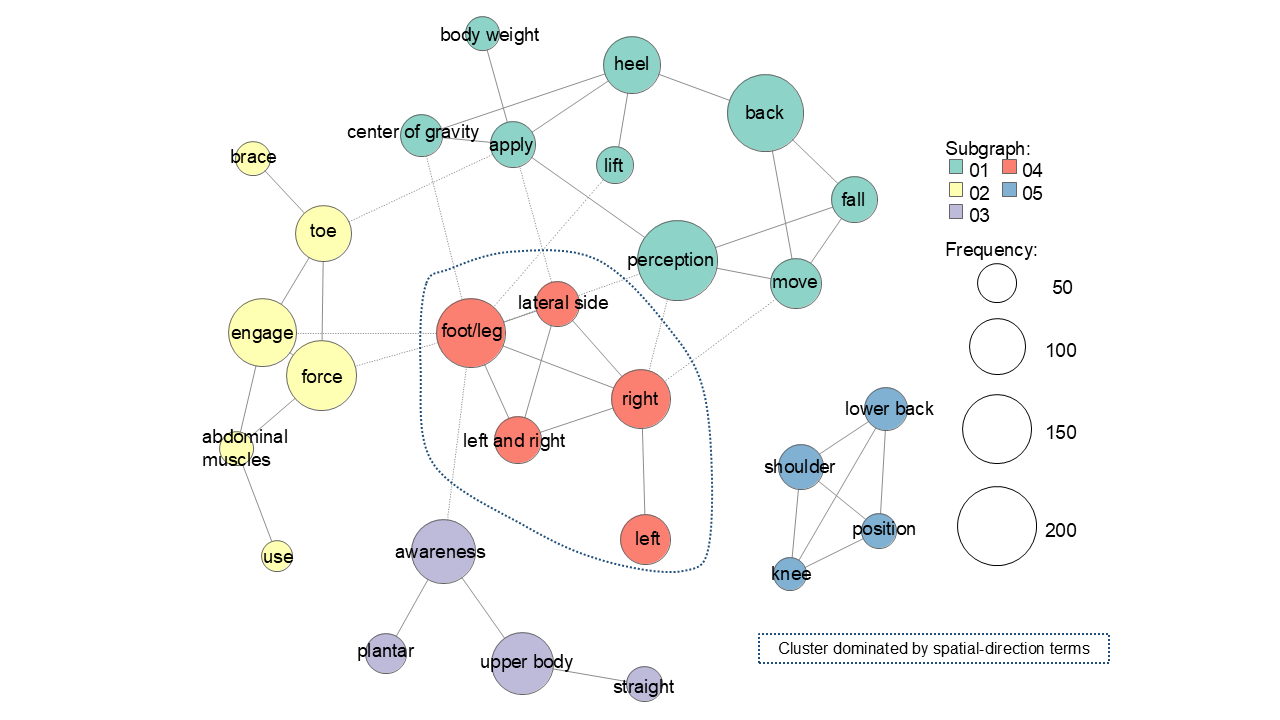

Supplement: Supplementary Figure S1 — Co-occurrence network of self-reported motor-strategy vocabulary when spatial-direction terms are retained. To illustrate the impact of retaining spatial-direction terms, verbal reports from all participants at T0 and T1 were pooled into a single corpus, and words with total frequency ≥25 in the pooled corpus were analyzed. Nodes represent words (size ∝ frequency), edges link words co-occurring within the same sentence, and colors represent data-driven subgraphs. For readability, we visualized the top 45 co-occurrence edges. When spatial-direction terms (e.g., “front,” “back/backward,” “left,” “right”) are retained, they co-occur strongly with foot-related terms and form a prominent direction/trajectory-centered cluster (dashed outline: Cluster dominated by spatial-direction terms), which can dominate the network structure under top-edge visualization and reduce interpretability for anatomical referencing. This figure is provided to justify excluding spatial-direction terms from the primary co-occurrence network (Figure 3). Abbreviations: T0, baseline; T1, immediately post-training [file Image1.tif]

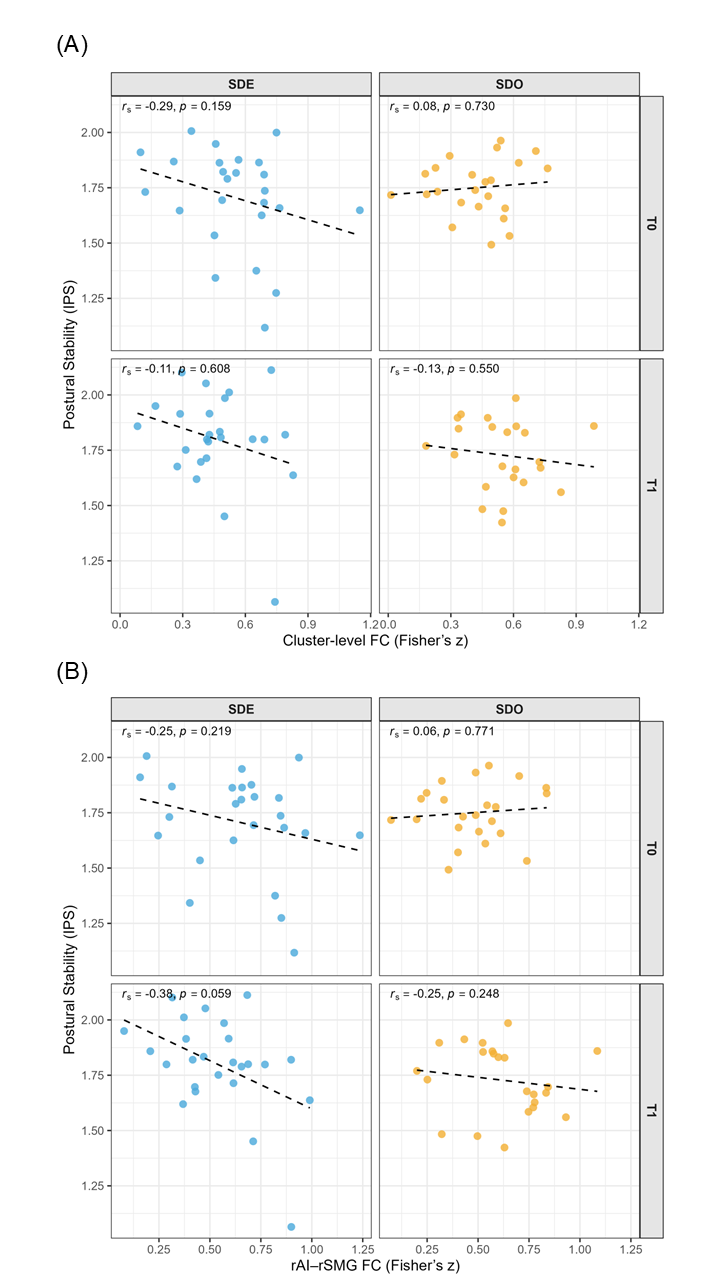

Supplement: Supplementary Figure S2 — Exploratory cross-sectional correlations between resting-state functional connectivity and postural stability at baseline and post-training. Scatter plots demonstrate the correlation between resting-state functional connectivity (Fisher's z; x-axis) and postural stability (IPS; y-axis) at T0 (baseline) and T1 (post-training), plotted by group. (A) Cluster-level FC (FC_cluster) of the TFCE-significant cluster. (B) Edge-level FC (FC_edge) of the connection that survived FDR correction (p-FDR < .05; right AInsula–right SMG). Points represent individuals, dashed lines indicate least-squares fits, and Spearman's rs and p-values are annotated. These analyses were conducted as exploratory, cross-sectional complements to the primary change-score correlations. FC, resting-state functional connectivity; IPS, index of postural stability; FDR, false discovery rate; SDE, sensory discrimination with expansion of attentional scope; SDO, sensory discrimination only; rs, spearman's rank correlation coefficient; rAI, right anterior insula; SMG, supramarginal gyrus; TFCE, threshold-free cluster enhancement; T0, baseline; T1, post-training. [file Image2.tif]
